# Neonatal lupus with complete heart block and long term follow up

**DOI:** 10.1093/omcr/omae056

**Published:** 2024-06-07

**Authors:** Ahmed Qasim Mohammed Alhatemi, Ali Akeel Al-Yacopy, Hashim Talib Hashim, Rand K Abdulhussain

**Affiliations:** Department of Internal Medicine, Al Nasiriyah Teaching Hospital, Thi Qar, Iraq; Coronary Care Unit, Al Nasiriyah Heart Hospital, Thi Qar, Iraq; College Of Medicine, Warith Al Anbiyaa University, Karbala, Iraq; Pharmacy Department, University of Huddersfield, Huddersfield, UK

**Keywords:** obstetrics/gynecology, critical care medicine, cardiology, emergency medicine, rheumatology

## Abstract

We present the case of a 27-year-old pregnant woman, newly diagnosed with Systemic Lupus Erythematosus (SLE) during pregnancy. The patient delivered a newborn at 38 weeks gestation, who, on the first day of life, manifested complete heart block. This case underscores the clinical challenges associated with neonatal lupus, emphasizing the need for collaborative, multidisciplinary management.

## INTRODUCTION

Neonatal Lupus with Complete Heart Block represents a poignant intersection of autoimmune pathology and its impact on fetal development. Neonatal Lupus is a rare condition arising from the transplacental passage of maternal autoantibodies, predominantly anti-Ro (SSA) and anti-La (SSB), to the developing fetus [1]. These autoantibodies, while often benign for the mother, can exert profound effects on the unborn child, with one of the most severe complications being the development of complete heart block [2].

This autoimmune-mediated cardiac conduction defect is characterized by an interruption of electrical signals between the atria and ventricles, resulting in a slow and irregular heart rhythm. The consequences of complete heart block are particularly critical in the neonatal context, where the fetal heart is at a crucial stage of development [3]. The fetal myocardium, under the relentless influence of the maternal autoantibodies, can experience significant damage, leading to impaired cardiac function and potential heart failure [3].

The introduction of the term ‘Neonatal Lupus’ underscores the temporal connection between the maternal autoimmune response and the neonate’s clinical presentation. It’s essential to recognize that while these infants may not exhibit overt symptoms of systemic lupus erythematosus (SLE), they bear the brunt of the autoimmune assault on their cardiac conduction system [4].

The link between anti-Ro and anti-La antibodies and the development of complete heart block in Neonatal Lupus has prompted extensive research into the underlying mechanisms. However, much remains to be elucidated about the precise pathways through which these antibodies interfere with fetal cardiac tissue [5]. Understanding these mechanisms is crucial for developing targeted interventions that could potentially mitigate the impact of Neonatal Lupus on the developing heart [6].

In this complex interplay between maternal autoimmunity and fetal cardiac vulnerability, Neonatal Lupus with Complete Heart Block emerges as a critical area of study and clinical concern [7]. As we delve deeper into the intricacies of this condition, the ultimate goal is to unravel the mysteries surrounding its pathogenesis and, in doing so, pave the way for innovative therapeutic strategies to safeguard the cardiac health of neonates born to mothers with autoimmune disorders [8].

## CASE REPORT

**Figure 1:**
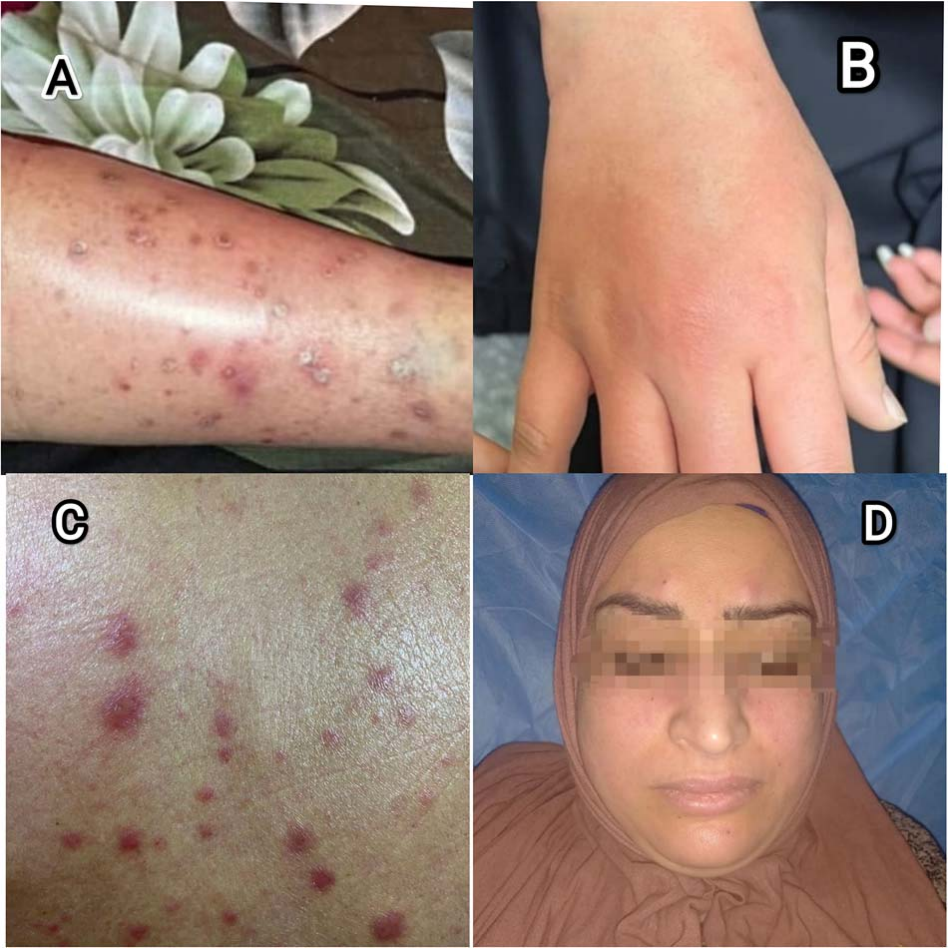
(**A**) Maculopapular on her shin. (**B**) Painful arthritis of the small joints of the hand with rash. (**C**) Maculopapular rash on her back. (**D**) Malar rash.

A 27-year-old primgravida in her Second trimester, sought care at the internal medicine outpatient clinic with a history of persistent non itchy maculopapular rash on her lower limbs and back, oral aphthous ulcer, arthritis and malar rash (Fig. 1) exacerbated by sunlight exposure and hot weather. Concurrently, she reported symmetrical and bilateral arthritis mainly affecting the knee and elbow joints and morning stiffness. Her symptoms started before one year and she visited multiple clinics for her persistent complaints with no clear diagnosis.

She is a non-smoker and non-drinker with no previous medical history. She was not taking any medications aside from pregnancy tonics and had no family history of SLE, autoimmune thyroid disease, or any other autoimmune disease.

Upon examination, she demonstrated an average build with a BMI of 24.8 Kg/m^2^, without pedal edema, and maintained normotension with normal vital signs. Abdominal examination indicated uterine height corresponding with the period of gestation. Further laboratory investigations (Table 1) including a complete blood count, immunological screen, and renal function tests were ordered and the results below.

**Table 1:** Maternal blood tests

Test	Results	Units	Normal value
Hemoglobin	11.9	g/dl	12–15
White blood cells	5.4	10^3^/microliter	4.1–9.5
Platelets	290	10^3^/microliter	165–415
ANA	>50	Index	6–10
Anti Ku	1.33	Index	6–10
Anti Mi-2	0.1	Index	6–10
Anti-nRNP	0.33	Index	6–10
Anti-scl-70	0.8	Index	6–10
Anti-jo-1	1.77	Index	6–10
Anti-smith	>50	Index	6–10
Anto-Ro-52	>50	Index	6–10
Anti-SS-B	>50	Index	6–10
Anti- cardiolipin Igm	20.6	Index	<12
Anti- cardiolipin Igg	46.9	Index	<12
Anti- Beta2- glycoprotein Igm	35.12	Index	<20
Anti- Beta2- glycoprotein Igg	5.6	Index	<20
B. Urea	33	Mg/dl	10–40
Serum creatinine	0.5	Mg/dl	0.5–1.1
C3	95	Mg/dl	83–192
C4	20	Mg/dl	15–75

The blood tests revealed high positive results for ANA, Anti Ro, Anti SS, Anti Smith Antibodies, Anti beta 2 Igg/Igm glycoprotein and anti cardiolipin Igm/Igg antibodies. Thereby establishing the diagnosis of systemic lupus erythematosus/Anti phospholipid syndrome.

In our department, we initiated treatment with Azathioprine 200 mg BD, Prednisolone 40 mg OD, Hydroxychloroquine 200 mg OD and aspirin 81 mg OD.

**Figure 2:**
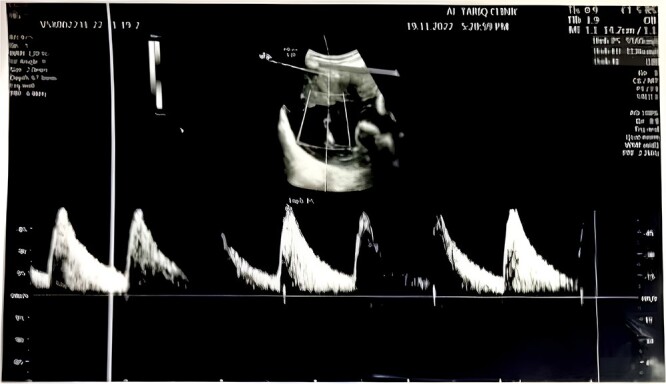
Neonatal Doppler ultrasound showing a single viable, active, and normal-tone female fetus in cephalic presentation and longitudinal lie. Gestational age: 31 weeks. Estimated fetal weight (EFW) = 1950 grams, with no sonographic signs of intrauterine growth restriction (IUGR). Normal amount of amniotic fluid and no gross fetal congenital abnormalities observed. Normal middle cerebral artery blood flow and spectral waveform. Biophysical profile = 8/8.

We continued to follow her up every two weeks during pregnancy with maternal Doppler ultrasound screening for any fetal anomalies or heart block (Fig. 2).

At the sixth trimester, we ceased aspirin and switched to Enoxaparin 6000 IU subcutaneous OD until delivery.

On the day of delivery, she underwent a cesarean section, delivering a full-term female baby weighing 2500 grams.

**Figure 3:**
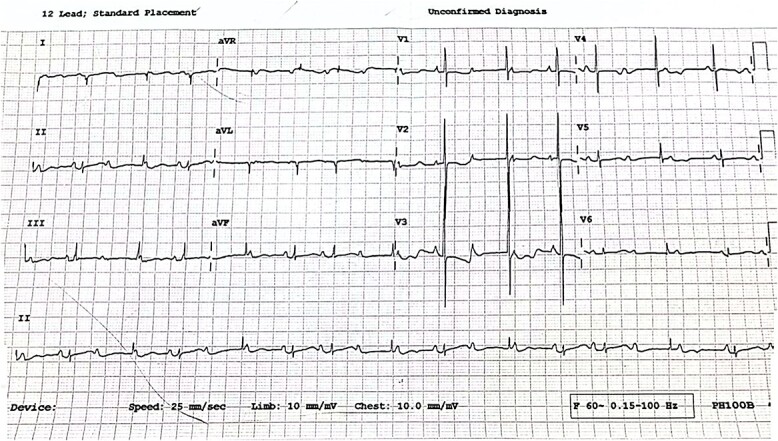
Neonatal ECG showing complete heart block with a heart rate of 78 beats per minute.

The neonate’s examination was unremarkable, with no manifestations of neonatal lupus erythematosus such as microcephaly, photosensitive rash, erythematous plaques, respiratory distress, or musculoskeletal abnormalities. The neonate remains stable in the ward with a respiratory rate of 33 cycles per minute, a rectal temperature of 36.7°C, and a heart rate of 78 beats per minute. An ECG was performed, revealing complete heart block (Fig. 3).

Blood tests, including a complete blood count, liver function tests, and renal function tests, were normal, showing no signs of cytopenia, elevated liver enzymes, or renal abnormalities. Arterial blood gas analysis and serum electrolyte levels of the baby were also within the normal range.

**Figure 4:**
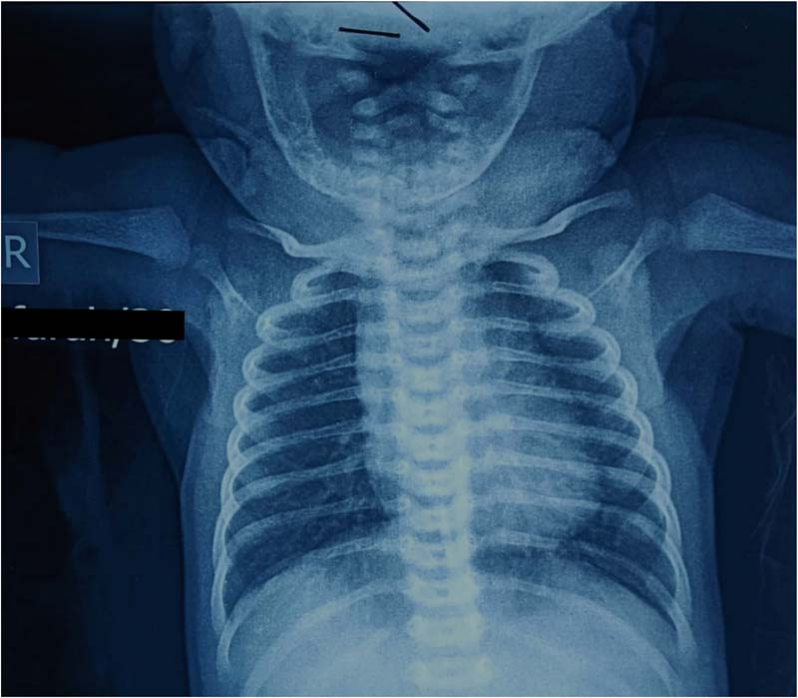
Neonatal chest X-ray PA view labelled as normal.

Echocardiography showed no structural abnormalities apart from mild pulmonary regurgitation. Left ventricular size and systolic function were normal. Chest X-ray was order and labelled as normal (Fig. 4).

Collaborative consultations with neonatology, cardiology, and rheumatology were undertaken. Pharmacological intervention included treatment with dexamethasone, with consideration of pacemaker placement if the baby develops severe bradycardia.

Following one week of delivery, both mother and neonate were discharged home without the need for a pacemaker, and a strict follow-up regimen was implemented for the mother and baby monthly for the first six months then every 3 months until 18 months has past since delivery.

No complications were noted and the baby did not develop severe bradycardia maintaining heart rate of 60–80 bpm.

Long-term follow-up plans included neonatal cardiology assessments, maternal lupus monitoring, and genetic counselling regarding the risk of recurrence in subsequent pregnancies.

## DISCUSSION

Neonatal Lupus, a rare autoimmune disorder, can have profound consequences when it presents with complete heart block in newborns. This condition arises from the transplacental passage of maternal autoantibodies, primarily anti-Ro and anti-La, to the developing fetus. While Neonatal Lupus typically manifests with cutaneous and cardiac manifestations, the occurrence of complete heart block is a critical concern [9].

Complete heart block in Neonatal Lupus results from the deposition of maternal autoantibodies within the fetal heart’s conduction system. This disruption impairs the electrical signals regulating heart rhythm, leading to a potentially life-threatening situation. The consequences are particularly dire because the fetal heart’s ability to compensate for such conduction abnormalities is limited [6, 8, 10].

Early detection and intervention are paramount in managing Neonatal Lupus with complete heart block. Treatment often involves a combination of medical therapies. One commonly used treatment is Hydroxychloroquine. Its administration during pregnancy shows promise for preventing recurrent congenital heart block in neonatal lupus erythematosus cases. This intervention could significantly impact prenatal care protocols for mothers with anti-Ro antibodies, potentially reducing the risk of this serious complication and improving neonatal outcomes [11]. In severe cases, pacemaker implantation shortly after birth is necessary. The pacemaker serves to regulate the heart’s rhythm, mitigating the risk of cardiovascular complications [10].

The current recommendations for follow-up of anti-Ro antibody-positive mothers include close prenatal monitoring, serial fetal echocardiograms, and postnatal clinical assessments to detect neonatal lupus erythematosus (NLE) manifestations. Long-term follow-up is also advised to monitor for delayed-onset NLE features. These measures aim to improve early detection and management, thus enhancing outcomes for both mothers and infants [12].

## CONCLUSION

Neonatal lupus with complete heart block presents significant challenges, necessitating multidisciplinary management and close monitoring. Promising interventions like Hydroxychloroquine offer hope for preventing recurrent heart block and potentially improving prenatal care protocols. Timely intervention, collaborative consultations, and long-term follow-up are crucial for optimizing outcomes for both mothers and infants. Further research is essential to advance understanding and enhance clinical strategies in addressing this complex condition.
